# Novel gnd_v2 Fusion Tag and Engineered TEV Protease Enable Efficient Production of Brazzein

**DOI:** 10.4014/jmb.2407.07047

**Published:** 2024-09-09

**Authors:** Yu Wang, Jiayao Zheng, Fan Wen, Bowen Tu, Lun Cui

**Affiliations:** 1 CCZU-JITRI joint Bio-X Lab, School of Pharmacy & School of Biological and Food Engineering, Changzhou University, 213164, Changzhou, Jiangsu Province, P.R. China; 2 Pathogenic Biological Laboratory, Changzhou Disease Control and Prevention Centre, Changzhou Medical Centre, Nanjing Medical University, 213000, Changzhou, Jiangsu Province, P.R. China

**Keywords:** gnd_v2 fusion tag, Tobacco Etch Virus (TEV) Protease, sweet protein brazzein, protein expression, ultrafiltration-based purification

## Abstract

Protein solubility and purification challenges often hinder the large-scale production of valuable proteins like brazzein, a potent sweet protein with significant health benefits and commercial potential. This study introduces two novel tools to overcome protein expression and purification bottlenecks: a gnd_v2 fusion tag and an engineered Tobacco Etch Virus (TEV) protease. The gnd_v2 tag, derived from 6-phosphogluconate dehydrogenase, was engineered to improve the soluble expression of brazzein. This tag increased brazzein's solubility by four times compared to the wild-type gnd tag, marking a significant advancement in efficient brazzein production. To address the challenge of cleaving the fusion tag, we engineered a TEV protease variant with high efficiency, particularly at the glutamine residue at brazzein's P1' site – a known difficulty for wild-type TEV proteases. We achieved streamlined production of pure, functional brazzein by integrating this tailored protease cleavage with an ultrafiltration-based purification protocol. Notably, the purified brazzein demonstrated a sweetness potency approximately 2500 times that of sucrose, highlighting its potential as a high-intensity natural sweetener. While this study focused on brazzein, the gnd_v2 tag shows promise for enhancing the solubility of other challenging proteins. More broadly, this work presents a versatile toolset for the scalable production of diverse functional proteins, with significant implications for industrial applications in food and pharmaceutical domains.

## Introduction

In the quest for healthier sweeteners [1-4], proteins that can replicate the sensory profile of sugar without its caloric content are emerging as promising substitutes. Proteins such as thaumatin (2000 times the sweetness of sucrose) [5, 6] and monellin (100000 times the sweetness of sucrose) [7] represent novel substitutes that, due to their intensely sweet taste at low concentrations, which can aid in efforts to reduce the detrimental health effects associated with overconsumption of sugar. Excess sugar consumption has been associated with multiple adverse health outcomes [8–10], including increased risk for cardiovascular disease, metabolic syndrome, type 2 diabetes, non-alcoholic fatty liver disease, and certain cancers. While the biological mechanisms underlying these associated diseases continue to be investigated, a scientific consensus has emerged that high sugar intake is a significant public health concern. Additional research is needed to characterize the dose-response relationships and delineate the causal pathways involved. Nonetheless, developing and implementing effective interventions to reduce sugar overconsumption represents an important priority for improving public health. Sweet proteins have recently been discovered to represent promising candidates for developing low-calorie sugar substitutes [1, 11]. Brazzein demonstrates a sweetness intensity several thousand times greater than sucrose and retains its sweetness when subjected to high temperatures (up to 80°C) [12, 13]. Isolated initially from the *Pentadiplandra brazzeana* plant, brazzein exhibits properties that make it a promising low-calorie natural sweetener. Its usage in the food system could aid public health efforts to curb the overconsumption of conventional sugars and associated adverse health issues.

While brazzein shows promise as a natural sweetener [14], its limited agricultural yields from the native *Pentadiplandra brazzeana* plant preclude the feasibility of large-scale production to meet global demand. Consequently, biotechnological methods have been explored to produce brazzein at scale. However, these methods, too, face hurdles. In yeast expression systems, the production levels of brazzein are low [15-17], while in bacterial systems like *E. coli*, the protein tends to form insoluble aggregates [18], complicating its recovery and purification. The limited solubility and purification challenges associated with brazzein have hindered its large-scale biotechnological production, creating a significant bottleneck for its commercialization. Developing innovative expression and production techniques is essential for overcoming the solubility and purification challenges that currently hinder the large-scale commercialization of brazzein, enabling higher yields of functional protein.

Fusion tags are a cornerstone of recombinant protein technology [19, 20]. They are utilized to facilitate expression levels and solubility of proteins by attaching to a target protein and aiding in its proper folding and stability. Despite their widespread use, the efficiency of these tags can vary significantly depending on the protein of interest. Tags like the small ubiquitin-like modifier (SUMO) [21] and the bacterial immunity factor B (infB)[22] have been used with varying degrees of success. For instance, while SUMO has been shown to enhance solubility and folding, its application in the expression of brazzein has led to suboptimal yields [23], underlining the necessity for more effective solutions. Our study introduces a fusion tag derived from the 6-phosphogluconate dehydrogenase (gnd) gene [24], a highly soluble protein that has yet to be previously exploited for this purpose. The gnd protein is known for its robust and high solubility properties, as shown in our previous study [25]. Our innovation lies in engineering this gnd tag, now called gnd_v2, which has been optimized to have fewer hydrophobic residues in two ends of the wild-type gnd protein, thus promoting its soluble expression. The optimized gnd_v2 tag presents a dual advantage: it increases the soluble expression levels of brazzein (2.7 times yield increase and 3.6 times soluble/insoluble ratio increase) and significantly simplifies the subsequent purification steps.

Complementing the gnd_v2 fusion tag, we have engineered a variant of the Tobacco Etch Virus (TEV) protease [26], specifically optimized to address cleavage efficiency challenge [27], especially accommodating the glutamine residue at the P1' site of brazzein. Integrating the novel gnd_v2 fusion tag with the optimized TEV protease alongside an ultrafiltration-based purification protocol culminates in a streamlined brazzein production pathway. This method solves the problems of solubility and purification without damaging brazzein's function. Our functional sweetness test showed that the purified brazzein is 2500 times sweeter than sucrose. This study proposes a novel approach to the scalable production of brazzein, addressing the critical bottlenecks and paving the way for its inclusion in a new generation of sweetening agents. The implications of our findings can extend well beyond the realm of sweet proteins, offering new avenues in the biomanufacturing sector for both food industry applications and therapeutic peptide production.

## Materials and Methods

### Bacteria and Growth Media

*E. coli* DH5α and *E. coli* BL21 (DE3) strains were sourced from New England Biolabs (USA). Luria-Bertani (LB) broth or LB agar from Sangon Biotech (China) was used for *E. coli* culture and growth. Bacteria containing the tags and brazzein expression plasmids were cultured in LB supplemented with 20 μg/ml of chloramphenicol.

### Plasmid Construction

All the plasmids were built based on the pACYCDuet-1 [28], which has the p15A origin, chloramphenicol-resistant genes, and two T7 promoters. The Gibson assembly method [29] was used for all the cloning. For the construction of plasmids pWY01-infB, pWY02-SUMO, and pWY03-gnd, primer LC368 and CYJ151 were used to linearize pACYCDuet-1 by polymerase chain reaction (PCR). The infB insert was PCR-amplified from the *E. coli* MG1655 genome with primer WY109 and WY120, and the SUMO fragment was generated from BBF10K_003422 T2T3_SUMO with primer WY111 and WY112 by using PCR. The wild-type gnd fragment was PCR-amplified from the *E. coli* MG1655 genome using primer WY113 and WY115.

For the construction of plasmids pWY04-his-infB-brz, pWY05-his-SUMO-brz, and pWY06-his-gnd-brz, the backbone and the brz fragments are shared. The backbone was the PCR product from pACYCDuet-1 using primer LC908 and CYJ151. The brz fragment was PCR-amplified by primer WY67 and CYJ150 from the synthesized brz DNA fragment (China). The infB fragment for pWY04-his -infB-brz was the PCR product from the *E. coli* MG1655 genome using primer WY88 and WY93. The SUMO fragment for pWY05-his -SUMO-brz was PCRed by using primer CYJ158 and WY97 from BBF10K_003422 T2T3_SUMO. The gnd fragment for pWY06-his -gnd-brz fragment was generated from the *E. coli* MG1655 genome using primer CYJ170 and WY68.

For the construction of the plasmid pWY07-gnd_v1-brz and pWY09-gnd_v2-brz, the plasmid backbone was the same as the one used for cloning pWY03-gnd, the brz fragment was the same as the one used for the cloning of pWY06-his -gnd-brz. The gnd_v1 fragment was generated with primer WY116 and WY68 from the *E. coli* MG1655 genome; the gnd_v2 fragment was PCRed from the *E. coli* MG1655 genome using primer WY116 and WY131. The cloning of pWY08-his-gnd_v2-brz was assembled from the pACYCDuet-1 backbone (used primer LC368 and CYJ151), the his-gnd_v2 fragment (used primer CYJ170 and WY1310) and the brz fragment (used WY67 and CYJ150).

The plasmid BBF10K_003570 (from BioBricks Repository) is used for the expression of wild-type TEV, the plasmid (pWY10-TEV-Max) for the expression of TEV Max was synthesized from Gentlegene (China). In both TEV constructs, the his-tag is located between the TEV cleavage site and the TEV gene, which results in auto-cleavage after the expression of the enzyme. *E. coli* DH5alpha was used for all the plasmid cloning. All the information of primes (Table S1) and synthesized gene fragments (Supplementary sequences S1 and S2) are provided.

### Protein Expression and Lysis

The BL21 (DE3) *E. coli* glycerol stock containing the expression plasmid was taken from storage at -80°C and used to inoculate 3 ml of Luria-Bertani (LB) supplemented with chloramphenicol. This starter culture was grown overnight (about 16 h) at 37°C with shaking at 180 rpm. The following morning, the culture was diluted into 20 ml fresh LB and grown at 37°C, 180 rpm until OD600 reached 0.5-0.6. Adding isopropyl β-D-1-thiogalactopyranoside (IPTG) to a final concentration of 0.5 mM for protein expression induction. Cultures were subsequently grown with 180 rpm shacking. The growth temperature could be 16°C, 25°C, or 30°C. The growth time can differ depending on the experiment design and requirement.

For lysis of the samples for further protein purification, centrifuge at 4,000 g for 10 minutes at 4°C, then resuspend the cell pellet with sample lysis buffer (50 mM Tris-HCl, 100 mM NaCl, 5 mM β-mercaptoethanol, pH 8.0). The resuspended mixture was then sonicated (50% amplitude, 2 s on, 2 s off, 5 min) on ice with a Scientz-IID ultrasonic homogenizer. The lysate was centrifuged at maximum speed to get the supernatant for purification.

### Sodium Dodecyl-Sulfate Polyacrylamide Gel Electrophoresis (SDS-PAGE) and Coomassie Staining

The protein samples lysed by centrifugation and sonication as described above. The soluble supernatants were removed, and pellets containing insoluble material were resuspended in 500 μl of inclusion body solubilization buffer (8 M urea, 50 mM Tris-HCl, 100 mM NaCl, 5 mM β-mercaptoethanol, 0.1% Tween-20, pH 8.0) followed by another round of sonication and centrifuge as described above.

For preparing gel loading samples, a solution containing 10 volumes of 2x Laemmli sample buffer obtained from Bio-Rad (USA), 1 volume of the chemical β-mercaptoethanol, and 9 volumes of the prepared soluble or insoluble samples. This prepared mixture was incubated at 95°C for 5 minutes. The samples that were prepared by mixing and incubation were then loaded into precast polyacrylamide gels containing 12% acrylamide obtained from Vazyme (China). These loaded gels were then subjected to electrophoresis using a Mini Gel Electrophoresis System instrument from Bio-Rad with the electrical voltage set at 80 volts for the stacking portion of the gel and 120 volts for the separating portion of the gel. Following the electrophoresis process, a standard Coomassie staining procedure was used to stain the gels, which were then imaged using the Gel Doc imaging system from Bio-Rad.

### Ni-NTA (nickel-nitrilotriacetic acid) Protein Purification

The cell lysate supernatant was filtered through a 0.22 μm filter to remove particles. His-tagged recombinant protein (TEV, TEV Max, His-gnd_v2-brz) was purified by using a Ni-NTA resin column equilibrated with 10 column volumes (CV) of binding buffer (50 mM Tris HCl pH 8.0, 50 mM NaCl, 5 mM β-mercaptoethanol, 0.1% Tween-20). The lysate was applied to the Ni column, then washed with 10 CV binding buffers. Then, it was washed with 10 CV wash buffer (50 mM Tris HCl pH 8.0, 50 mM NaCl, 5 mM β-mercaptoethanol, 0.1% Tween-20, 10 mM imidazole) to remove nonspecifically bound contaminants. Bound His-tagged protein was eluted with 4 CV of elution buffer (50 mM Tris HCl pH 8.0, 100 mM NaCl, 5 mM β-mercaptoethanol, 0.1% Tween-20, 250 mM imidazole) and collected in 1 CV fractions. An aliquot of each fraction was analyzed by using SDS-PAGE gels and stained with coomassie stain blue dye to assess protein purity. The above purification procedures were performed using an AKTA Explorer 100 system from GE Healthcare (USA).

### Tobacco Etch Virus (TEV) Protease Cleavage

To test the efficiency of TEV cleavage, purified recombinant protein (his-gnd_v2-brz) was incubated with His-tagged Tobacco Etch Virus (TEV) protease (TEV or TEV Max) at specific protein to TEV protease mass ratio (for TEV, the ratio is 5:1; for TEV Max, the ratio is 15:1) in cleavage buffer (50 mM Tris-HCl pH 8.0, 50 mM NaCl) [30]. The cleavage reaction was incubated at 4°C for 16 h (or 96 h) with gentle agitation. The separation of the cleaved brazzein protein from the gnd_v2 tag was confirmed by SDS-PAGE analysis.

### Cleavage of gnd_v2-brz Fusion Protein with TEV Max Protease and Subsequent Ultrafiltration

TEV protease (TEV Max) was purified by immobilized metal affinity chromatography using Ni-NTA agarose resin, as previously described. The gnd_v2-brz fusion protein expressed in *E. coli* was lysed in buffer containing 50 mM Tris-HCl, 100 mM NaCl, 5 mM β-mercaptoethanol, pH 8.0, and used for cleavage reactions. Cleavage was performed by incubating the fusion protein with TEV Max protease at a mass ratio of 15:1 fusion protein: protease. Reactions were incubated at 4°C for 16 h with gentle agitation.

Following cleavage, the reaction mixture was subjected to two sequential ultrafiltration steps to isolate brazzein from the cleavage products [31]. First, the sample was purified using a 10 kDa molecular weight cut-off (MWCO) spin filter to retain the gnd_v2 tag and TEV Max while allowing brazzein to flow through. The flow through brazzein was collected and purified again with 10 kDa MWCO spin filter to remove any remaining impurities. The flow through was further concentrated with 1 kDa MWCO spin filtrer. SDS-PAGE analysis was conducted on samples collected pre- and post-cleavage and after each ultrafiltration step. Gels were stained with coomassie stain blue, and gel images were taken using Geldoc. Cleavage efficiency was determined by densitometric analysis of the fusion protein and brazzein bands by using ImageJ software (1.54d). For preparing brazzein sample for tasting test, brazzein was dialyzed to ddH_2_O.

### Bradford Assay

The purified brazzein protein concentrations were quantified using the Detergent Compatible Bradford Protein Assay Kit produced by Vazyme, Nanjing, Jiangsu, China. The quantification was performed by first generating a standard curve using bovine serum albumin (BSA) across a range of concentrations from 0.025 milligrams per milliliter to 0.125 milligrams per milliliter. The brazzein protein samples containing differing amounts of sodium chloride were mixed with 1 milliliter of the Bradford reagent supplied in the kit for quantification. After mixing, 200 microliters from each mixture were transferred into the wells of a transparent 96-well plate suitable for absorbance measurements. The absorbance was read at a wavelength of 595 nanometers utilizing a Tecan Infinite 200 PRO Microplate Reader instrument. The concentrations of the proteins in the brazzein samples were calculated based on the bovine serum albumin (BSA) standards.

### Sweet Sensory Evaluation

A sweet sensory evaluation method was conducted to assess the sweetness intensity of brazzein. Simply, a panel of 22 untrained tasters were selected to taste 6 samples. Four Brazzein samples were prepared at 1, 1.5, 2, and 5 μg/ml concentrations, while two control sucrose samples were prepared at 3 and 5 mg/ml, serving as established benchmarks for sweetness perception [32, 33]. All 6 samples (marked A, B, C, D, E, and F, without correlation to the reagent concentration) were prepared using deionized water and presented to participants in a randomized order to prevent bias. Participants were instructed to taste each of the 6 samples, cleansing their palates with water between samples to avoid carry-over effects. The perceived sweetness of each solution was rated on a discrete scale: a negative score for non-sweet perceptions, and positive scores (represented as '+', '++', '+++', '++++', '+++++', or '++++++') for increasing sweetness intensity relative to other samples. Data collection involved recording the number of panelists detecting sweetness in each sample and their corresponding sweetness intensity scores. Cumulative sweetness scores were calculated to analyze the dose-dependent sweetness perception of brazzein. A comparative analysis between brazzein and sucrose was conducted to determine the relative sweetness of brazzein on a per mass basis. This comprehensive sensory evaluation method facilitated the quantification of brazzein's sweetness perception in relation to sucrose. This sweet sensory evaluation, involving Brazzein protein tasting by participants, has been thoroughly reviewed and approved by the Ethical Committee of Changzhou University, under the approval number CCZU-ER20231009.

### Alphafold2 Structure Modeling

A Google colab notebook (link below) was used for AlphaFold2 [32] and MMseqs2 [33] based structure modeling for the protein structure of gnd, gnd_v2-brz. A default setting was used for the structure modeling.(https://colab.research.google.com/github/sokrypton/ColabFold/blob/main/AlphaFold2.ipynb) [34].

### Data Analysis

Data were obtained from at least three independently repeated experiments, and all variables are expressed as mean ± standard deviation (SD).

## Results and Discussion

### Comparative Analysis of Fusion Tag Efficacy on Brazzein Solubility

Our previous study revealed that the 6-phosphogluconate dehydrogenase (gnd) gene can be expressed in a highly soluble form in *E. coli* [25]. Inspired by the hight solubility property, we explored the gnd genés potential as a fusion partner for enhancing the solubility of recombinant proteins. We compared two commonly utilized fusion tags-translation initiation factor IF-2 (infB) and Small Ubiquitin-related Modifier (SUMO)-with gnd for their ability to increase the soluble expression of brazzein, a new type of sweet protein. The three tags were expressed in *E. coli* both independently and in fusion with brazzein.

**Fig. 1:**
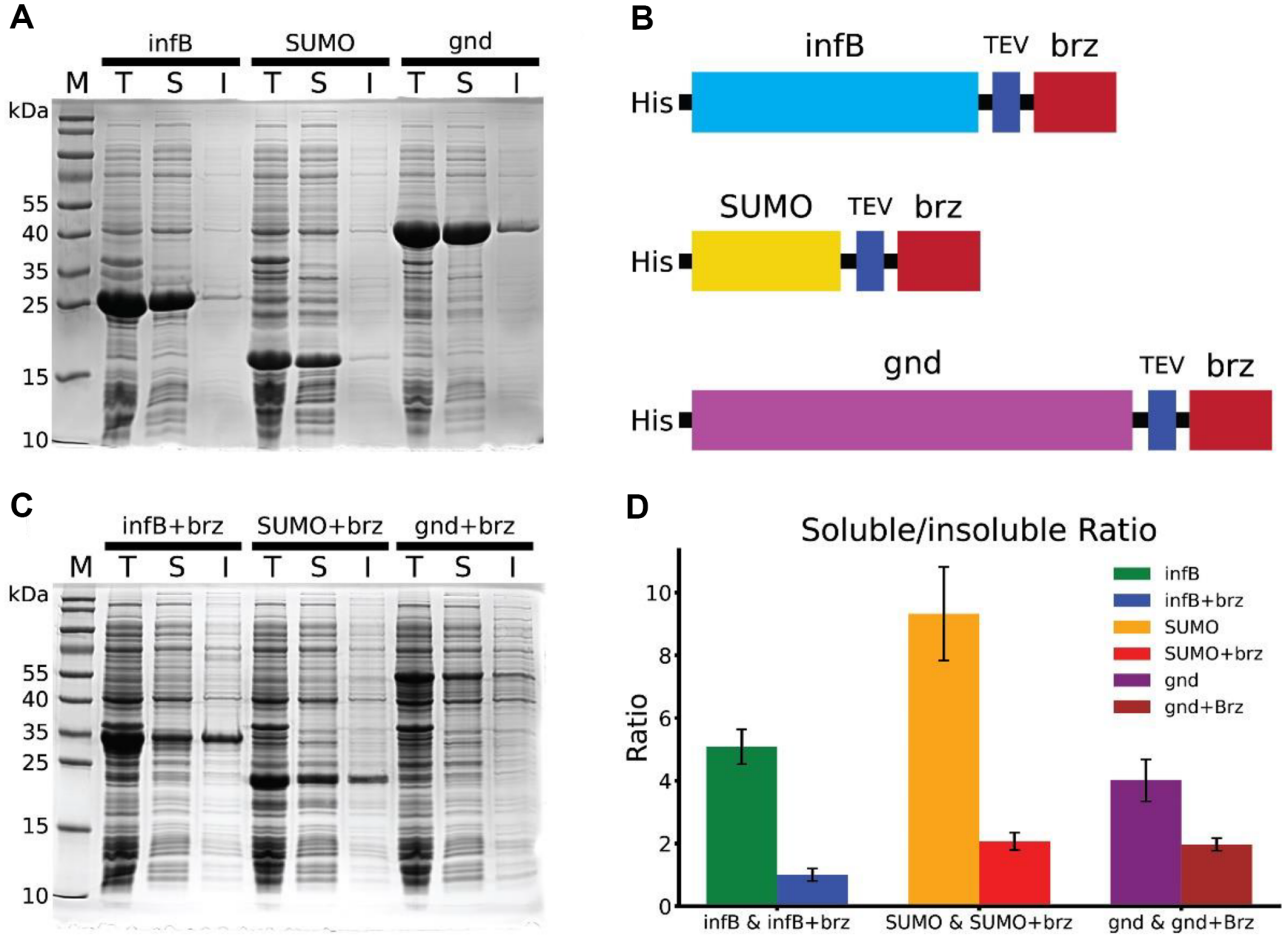
Solubility Analysis of Brazzein with Various Fusion Tags. (**A**) SDS-PAGE analysis of *E. coli* expressing infB, SUMO, and gnd tags without brazzein. The total protein (T), soluble (S), and insoluble (I) fractions are shown, along with molecular weight markers (M) for reference. (**B**) Diagram of the brazzein fusion constructs: His-infB-brazzein, His-SUMObrazzein, and His-gnd-brazzein, including the TEV protease cleavage site. (**C**) SDS-PAGE analysis of *E. coli* expressing brazzein fused with infB, SUMO, and gnd tags. (**D**) Comparison of soluble/insoluble protein ratios for the three tags, both with and without brazzein. Data are shown as mean ± standard deviation, with *n* = 3.

The Sodium dodecyl-sulfate polyacrylamide gel electrophoresis (SDS-PAGE) result presented in Fig. 1A demonstrated the intrinsic solubility of the tags in the absence of brazzein. SUMO exhibited the highest soluble/insoluble ratio of 9.3 (Fig. 1D). The ratios for infB and gnd were 5.1 and 4.0 (Fig. 1D), respectively. Despite gnd possessing the lowest ratio among the tags tested, we proceeded to construct expression vectors fusing the gnd tag to the N-terminus of brazzein with a Tobacco Etch Virus (TEV) cleavage site in between (Fig. 1B), based on its inherent solid solubility. Fig. 1C, showcasing the solubility profiles with the brazzein fusion, reveals a decrease in the soluble/insoluble ratio across all three tags. His-infB-brazzein constructs displayed an equal distribution between soluble and insoluble fractions, with a soluble/insoluble ratio of 1.0 (Fig. 1D). The fusion with SUMO and gnd resulted in similar soluble/insoluble ratios of 2.1 and 2.0, respectively (Fig. 1D). The gnd tag's comparable efficacy to the well-established SUMO tag in enhancing brazzein solubility is not apparent. The use of gnd, despite its initially lower soluble/insoluble ratio, suggests that the intrinsic properties of fusion partners should be considered in the context of their interaction with the target protein. When the gnd tag was used for brazzein expression, it achieved a soluble/insoluble ratio of 2.0, which is similar to the ratio of SUMO-brz, 2.1. This underlines the potential of exploring its utilization for solubility enhancement in brazzein expression.

In conclusion, the gnd fusion tag has demonstrated a capability to enhance the solubility of brazzein similar to that of the SUMO tag, despite gnd's lower intrinsic solubility ratio. The study provides a foundation for reconsidering the selection criteria for fusion tags and highlights the potential of gnd as a valuable tool for improving protein solubility in biotechnological applications.

### The gnd_v2 Variant increases Brazzein Solubility and Expression

**Fig. 2:**
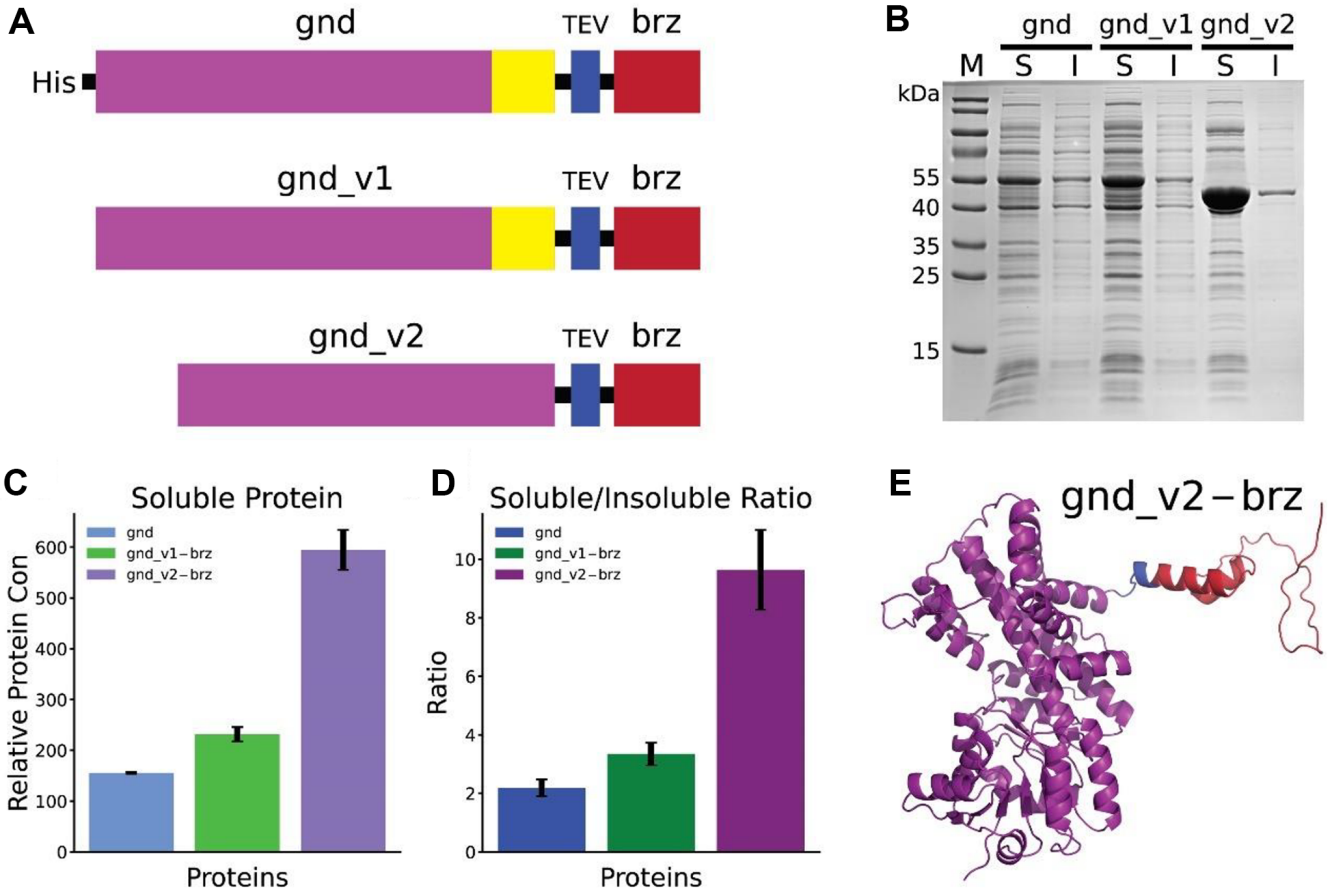
Enhanced Soluble Expression of Brazzein Using Optimized gnd_v2 Fusion Tag. (**A**) Schematic representation of brazzein (brz) fusion constructs with histidine-tagged gnd, gnd_v1, and gnd_v2. Each construct includes a TEV protease cleavage site (blue). (**B**) SDS-PAGE analysis comparing the solubility of brazzein when fused to original gnd, gnd_v1, and gnd_v2. 'M' represents the molecular weight marker, 'S' is the soluble protein fraction, and 'I' is the insoluble protein fraction for each construct. (**C**) Bar graph showing the relative concentration of soluble brazzein for each fusion construct, normalized to protein marker. Error bars indicate standard deviation from triplicate experiments. (**D**) Bar graph representing the soluble/insoluble ratio for brazzein when expressed with each fusion tag. (**E**) Alpahfold2 predicted tertiary structure of brazzein fused to gnd_v2, with gnd_v2 in purple, TEV cleavage site in blue and brazzein in red.

To enhance the solubility of the sweet protein brazzein, we have developed two variants of the 6-phosphogluconate dehydrogenase (gnd) fusion tag: gnd_v1 and gnd_v2. The modifications were designed to further improve the solubility of gnd tag. The initial construct, his-gnd-brz, included an N-terminal histidine (His) tag, which is known for its relative hydrophobicity and potential to reduce protein solubility [35, 36]. Structural modeling (Fig. S1) suggested that the surface exposure of His tag might adversely impact solubility. In response, we deleted the N-terminal His tag, resulting in the gnd_v1 variant (Fig. 2A). Subsequent analysis indicated that the C-terminal segment of gnd was identified (Fig. S1 in yellow color) and consisting of a hydrophobic sequence (VLPANLIQAQRDYFGAHTYKRIDKEGVFHTEWLD) may contribute to aggregation and insolubility due to the propensity of hydrophobic amino acids to aggregate in aqueous solutions [37]. The predicted model also showed that the position of the C-terminal segment is disruptive to the whole protein's globular structure [38, 39], which can result in decreased solubility. To mitigate this, the segment was removed to create the gnd_v2 variant (Figs. 2A and S2), hypothesizing that these modifications would enhance solubility by having a more hydrophilic and globular structure.

The SDS-PAGE analysis provided a comparative assessment of the solubility profiles of brazzein expressed with each gnd variant (Fig. 2B). The gnd_v2 variant demonstrated a significant increase in soluble protein expression, evidenced by the enhanced band intensity in the soluble fraction and a corresponding decrease in the insoluble fraction, compared to the original gnd and gnd_v1 variants. Quantitative analysis confirmed a 2.7-fold enhancement in soluble protein yield with the gnd_v2 variant relative to the original gnd tag (Fig. 2C). This observation was further supported by the soluble/insoluble ratio data (Fig. 2D), which showed a markedly 3.6-fold soluble/insoluble ratio increase for gnd_v2 fused with brazzein. This high gnd_v2-brz soluble/insoluble ratio (9.64) is similar to that of the SUMO tag alone (9.32), signifying the superiority of the gnd_v2-brz soluble expression. The structural illustration (Fig. 2E) shows the predicted structure of brazzein fused with the gnd_v2 tag, substantiating the solubility enhancement noted experimentally. The augmented solubility and expression achieved with the gnd_v2 variant are attributed to its refined ability to support correct protein folding and reduce aggregation tendencies, which are paramount for the recovery of biologically active recombinant proteins.

The development of gnd_v2 represents a significant advancement in engineering the tag to improve the brazzein solubility challenge. The removal of hydrophobic residues in both the N-terminal and C-terminal of gnd itself can improve protein solubility, and it also makes the gnd central part more globular, which is critical for the solubility of proteins. Our study showed that the engineered gnd_v2 tag achieved doubled protein yield and about 4 tiems of soluble/insoluble ratio. Moreover, the engineered gnd_v2 is also 5 Kb smaller than the original gnd tag. This result emphasizes the importance of structural considerations in the design of fusion tags and showcases the potential for custom-designed tags to improve protein production outcomes.

### Enhanced Efficiency of Engineered TEV Protease Variant (TEV Max) for Targeted Cleavage at P1' Glutamine

**Fig. 3:**
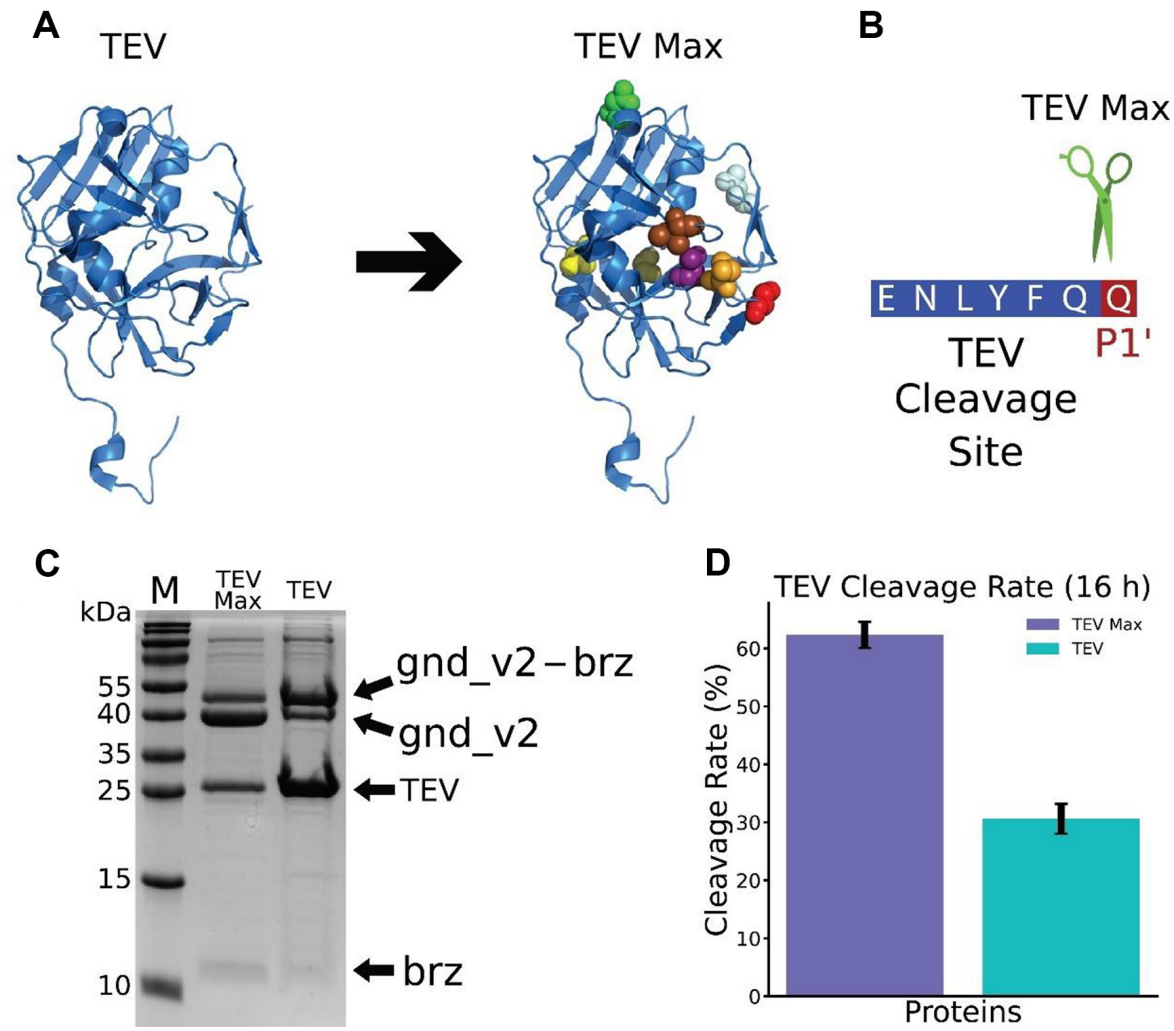
Enhanced TEV Protease Variant (TEV Max) for Targeted Cleavage at P1' Glutamine. (**A**) Structural illustration of wild-type TEV protease (left) and TEV Max (right). The nine mutations introduced in TEV Max are highlighted with sphere shape in different colors. (**B**) Illustration of the TEV Max cleavages at the TEV cleavage site. The P1’ position is highlighted in red. (**C**) SDS-PAGE analysis of His-gnd_v2-brazzein cleavage by TEV Max and wild-type TEV protease. 'M' represents the molecular weight marker, 'TEV Max' is the result of TEV Max cleavage, and 'TEV' is the result of TEV cleavage. The ‘brz’ marks the position of the brazzein position in the gel. (**D**) Quantification of brazzein cleavage by TEV Max and wildtype TEV protease after 16 h incubation at 4°C. Error bars represent standard deviation (*n* = 3).

The efficient release of brazzein from the gnd_v2 tag is a critical step in producing this high-potency sweetener. Tobacco Etch Virus (TEV) protease was utilized to cleavage the fusion protein due to its highly specific recognition sequence and ease of recombinant production in *E. coli*. However, the typical TEV protease has low cleavage efficiency at a glutamine residue at the P1' site [27], a bottleneck for proteins like brazzein that contain this specific amino acid at the first residue of N-terminal. To circumvent this limitation, we engineered an enhanced TEV protease variant, named TEV Max, through rational mutagenesis based on previous extensive studies to improve enzyme stability and broaden substrate specificity [40-44]. Nine mutations (T17S, L56V, N68D, I77V, S135G, I138T, S153N, T180A, V219N) were strategically introduced to improve enzyme stability, broaden substrate specificity, and optimize catalytic efficiency. Mutations such as T17S, L56V, and I77V were selected to enhance structural stability, essential for maintaining activity under various conditions, while S135G, I138T, and S153N were targeted to expand substrate specificity, particularly to accommodate glutamine at the P1' site. Additionally, modifications like N68D, T180A, and V219N were included to increase the enzymés catalytic turnover rate. Structural and kinetic analyses guided these selections, ensuring that each mutation contributed to overcoming the wild-type enzymés limitations. As shown in Fig. 3A, these mutations are strategically located to enhance TEV Max’s performance, resulting in a uniform cleavage profile across all 19 amino acids at the P1' site (Figs. 3B and S3), a substantial improvement over the wild-type enzyme, , which only preferentially cleaves at serine (S), glycine (G), alanine (A), methionine (M), cysteine (C), or histidine (H). Thus, TEV Max represents a robust solution for the efficient release of brazzein, with a design grounded in a thorough understanding of TEV protease’s structural and functional determinants.

In Fig. 3C, we present a direct comparison of the cleavage efficiency of His-gnd_v2-brz by TEV Max and the wild-type TEV protease. The experiment required the addition of a two-fold excess of the wild-type enzyme due to its lower efficacy; otherwise, the cleavage of brazzein by wild-type TEV was not detectable when equal amounts were used. The SDS-PAGE analysis demonstrates that, after 16 hours of incubation at 4°C, the TEV Max-treated sample exhibited a pronounced increase in the band corresponding to cleaved gnd_v2 tag, indicative of better proteolysis relative to the wild-type TE, even at 2 times higher enzyme concentrations. Quantitative analysis (Fig. 3D) confirms TEV Max's enhanced efficiency, achieving 62.3% cleavage compared to 30.6% with the wild-type TEV. Notably, an extended incubation period of 96 hours at 4°C resulted in increased cleavage efficiency for both TEV variants (89.3% for TEV Max and 70.2% for wild-type TEV, Fig. S4). However, TEV Max still maintained a higher efficiency throughout, underscoring the success of our protein engineering strategy.

Our study chooses TEV as the enzyme for releasing brazzein from fusion proteins, but the P1’ site in the gnd_v2-brz construct is glutamine, an unfavorable amino acid for wild-type TEV enzyme. An MPB-TEV structure was built with 9 cumulative mutations in the TEV sequences [45]. To our surprise, our TEV Max enzyme can have the same cleavage efficiency for all 19 amino acids. The only amino acid that showed little cleavage is proline, which can also not be cleaved by wild-type TEV. The engineering of TEV Max to overcome specific cleavage challenges is not for brazzein only because it has high cleavage efficiency for all 19 different amino acids. This result validates the engineered TEV Max as a highly effective proteolytic tool for processing fusion proteins with glutamine at the P1' site. The increased P1’ site cleavage spectrum of TEV Max enable the broaden application of TEV cleavage for recombinant protein production.

### Streamlined Integrative Process for Purification of Brazzein with ultrafiltration

**Fig. 4:**
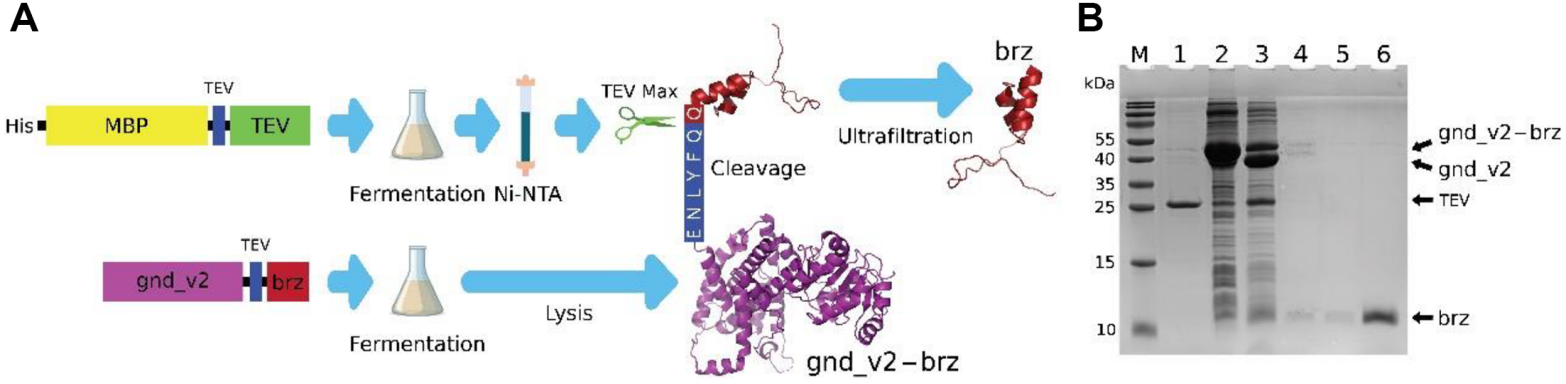
Streamlined Purification of Brazzein via Ultrafiltration and Specific Cleavage by TEV Max Protease. (**A**) Schematic overview of the purification process. It begins with the fermentation and Ni-NTA purification of the TEV Max; then it is mixed with lysed gnd_v2-brazzein. The gnd_v2-brz fusion protein is cleaved by the purified TEV Max protease, and the reaction mixture is processed through sequential ultrafiltration steps with 10 kDa and 10 kDa MWCO filters to get highpurity brazzein. (**B**) SDS-PAGE analysis of the purification process. Lane M: Molecular weight marker; Lane 1: Purified TEV Max protease; Lane 2: Supernatant of gnd_v2-brazzein lysis; Lane 3: Mixture post-cleavage by TEV Max protease after 16 h of incubation with gnd_v2-brz; Lane 4: Filtrate post-10 kDa MWCO ultrafiltration; Lane 5: Final purified brazzein post-10 kDa MWCO ultrafiltration; Lane 6: Concentrated brazzein of the 10 Kda ultrafiltration product.

This phase of our study delineates an integrated approach for the purification of brazzein, which involves the use of the engineered gnd_v2 fusion tag (for expressing brazzein) and subsequent cleavage by the optimized TEV Max protease, as showed in Fig. 4A. The process begins with the expression of brazzein as a fusion protein with the gnd_v2 tag to enhance solubility and yield. Following fermentation, the cells are harvested by centrifugation and lysed via sonication to release the gnd_v2-brazzein fusion protein into the supernatant.

The fusion protein supernatant is then incubated with Ni-NTA (nickel-nitrilotriacetic acid) purified TEV Max protease to facilitate selective cleavage, releasing brazzein. After 16 h of incubation at 4°C, the mixture is first subjected to ultrafiltration with a 10 kDa molecular weight cut-off (MWCO) filter, which allows smaller fragments, brazzein, to pass through and results in 84% purity (Fig. S5). Further refinement is achieved through a subsequent ultrafiltration step using a 10 kDa MWCO filter. This stage removed big molecular weight contaminants and achieved 96% purity (Fig. S5). The SDS-PAGE analysis in Fig. 4B presents the sequential purification steps: Lane 1 shows the purified TEV Max protease; Lane 2 displays the supernatant from the gnd_v2-brazzein lysis; Lane 3 reveals the mixture post TEV Max cleavage; Lanes 4 and 5 illustrate the protein profiles following sequential ultrafiltration steps, with Lane 5 displaying a clear band corresponding to the purified brazzein; Lane 6 exhibits the final concentrated and purified brazzein product.

The integration of optimized expression and cleavage strategies with a simplified ultrafiltration-based purification protocol demonstrates the feasibility of streamlining protein purification processes. The simplification of the purification is based on: (1) The high-level expression of soluble brazzein. Utilizing the gnd_v2 tag significantly increases the solubility and expression level of brazzein, resulting in a substantial portion of the target protein being present in the supernatant post-cell lysis. The employment of the gnd_v2 tag in flask fermentation of gnd_v2-brz optimizes the yield of soluble target protein, facilitating easier downstream processing. (2) The high cleavage efficiency of TEV Max. The TEV Max protease demonstrates exceptional cleavage efficiency, overcoming the limitations associated with the P1 sités restriction. This efficiency allows for the direct mixing of Ni-NTA purified TEV Max with the crude, unpurified gnd_v2-brz extract in a straightforward buffer solution (comprising 100 mM Tris HCl and 50 mM NaCl). The result is a highly efficient cleavage process that does not necessitate extensive preparation or purification of the reactants. (3) The relatively small size of brazzein. Brazzein's relatively small molecular weight (6.5 kDa), which is less than that of most proteins in the cleavage mixture, significantly contributes to the simplification of the purification process. Employing consecutive ultrafiltration steps (first with a 10 kDa, repeated with a 10 kDa) effectively separates brazzein, yielding a highly purified product. This method leverages brazzein's unique size to streamline the purification process, reducing the need for more complex and time-consuming techniques.

This integrative purification strategy, employing a combination of affinity chromatography and two-stage ultrafiltration, results in a highly pure brazzein purification. This streamlined bacteria-to-brazzein protocol makes it feasible for large-scale industrial production because of its simplicity and low cost. Even if the high-cost Ni-NTA purification method was used for TEV Max purification, all other purification steps are matured at low prices in industrial applications.

### Sensory Evaluation Demonstrates Dose-Dependent Sweetness of Brazzein

A randomized sweet sensory evaluation method was conducted with a panel of 22 tasters to evaluate the sweet perception of brazzein's sweetness [32], and sucrose samples were used as benchmarks for sweetness perception [33].

**Fig. 5:**
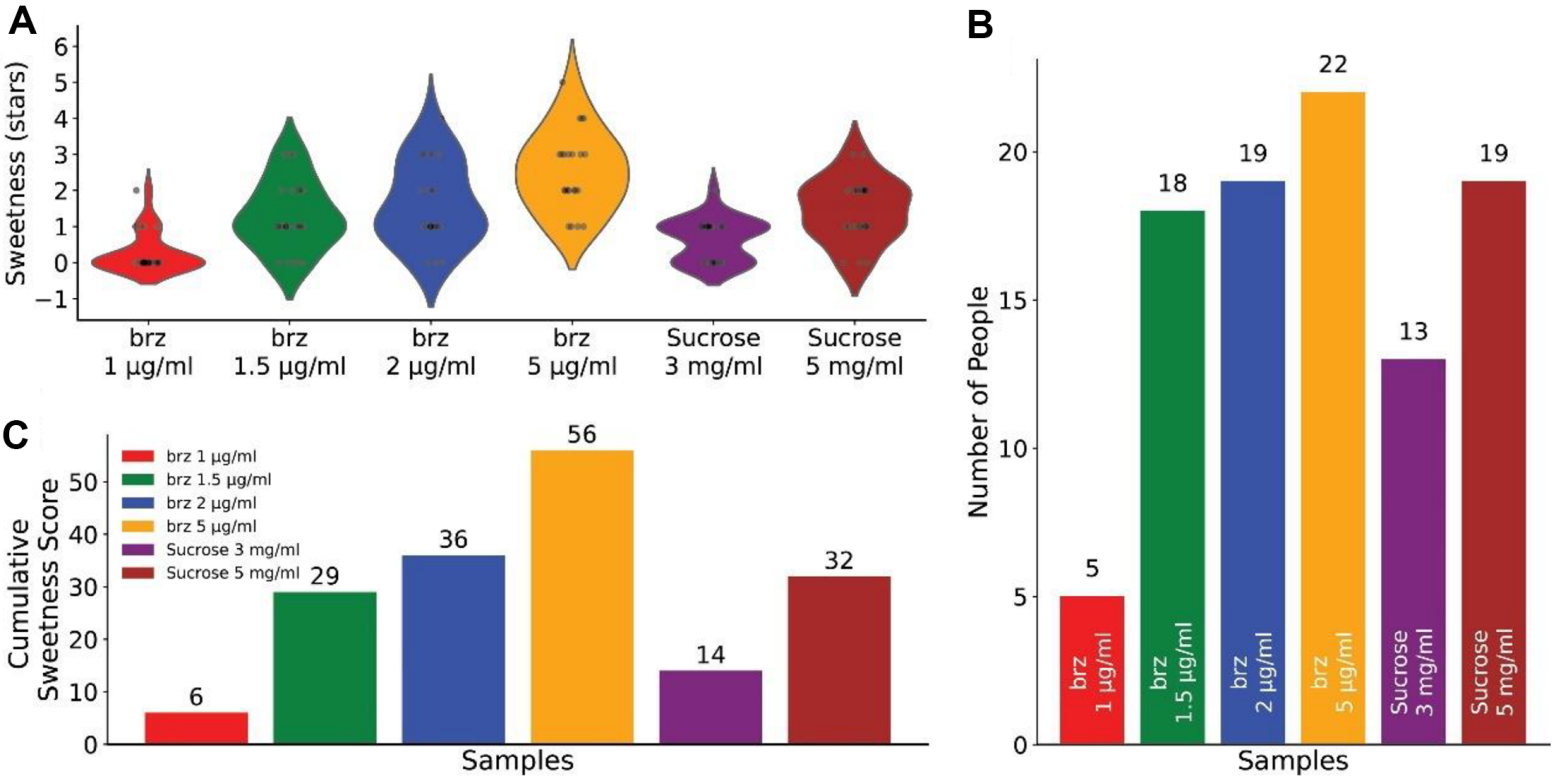
Sensory Evaluation of Brazzein Sweetness. (**A**) Violin plots depicting the distribution of sweetness scores for various concentrations of brazzein and sucrose. Each plot represents the range of scores, with individual points indicating scores from each taster. (**B**) Bar chart illustrating the number of participants who rated each solution as sweet. (**C**) Bar chart illustrating the cumulative sweetness socores of each samples.

Fig. 5A shows a dose-dependent increase in perceived sweetness was observed for both brazzein and sucrose. At the lowest tested concentration of brazzein (1 μg/ml), 5 people detected it as sweet, with only one individual assigning a score of two '+' marks. In contrast, at a slightly higher concentration of 1.5 μg/ml, the majority (*n* = 18) recognized the solution as sweet, surpassing the sweetness detection rate of sucrose at 3 mg/ml, rated as sweet by 13 participants. At a brazzein concentration of 2 μg/ml, there were still three participants who did not perceive the solution as sweet. However, this concentration yielded a cumulative sweetness score (Fig. 5C) comparable to that of sucrose at 5 mg/ml, the latter being a concentration commonly cited as the human sweetness threshold for sucrose. This observation suggests that the sweetness threshold for brazzein is approximately 2 μg/ml, implying that brazzein is roughly 2500 times sweeter than sucrose on a per mass basis. Notably, all tasters found the 5 μg/ml brazzein solution to be sweet and received high sweetness scores across the board (Fig. 5B). Fig. 5B presents the number of positive responses for each solution. The sweetness scores across different concentrations of brazzein and compared to sucrose support the factor of brazzein's significantly heightened sweetness. These findings validate the use of brazzein as a potent sweetener, with promising applications in food and beverage formulations where sugar reduction is desired without compromising taste.

## Conclusion

This study comprehensively explores the enhancement of solubility, expression, and purification processes for brazzein, a high-intensity natural sweetener, addressing significant challenges in its biotechnological production. Our results demonstrate the successful application of the gnd_v2 fusion tag to significantly improve brazzein solubility and yield in *E. coli*. Comparative analyses highlighted the superior performance of gnd_v2 over traditional tags such as SUMO and infB. Additionally, the engineered TEV Max protease variant exhibited enhanced efficiency in cleaving TEV cleavage site coupled brazzein, optimizing the purification process. The streamlined purification protocol employing ultrafiltration facilitated the recovery of high-purity brazzein. Sensory evaluations confirmed brazzein's dose-dependent sweetness, significantly surpassing that of sucrose and reaffirming its potential as a potent, low-calorie sweetener.

We have laid the groundwork for its industrial production as a natural, low-calorie sweetener by addressing key challenges in the expression, solubility, and purification of brazzein. To advance the commercialization of brazzein and other potentially sweet proteins, future research should prioritize scaling up production, applying these techniques to additional proteins, and assessing the long-term health implications of brazzein consumption. Furthermore, exploring alternative sweet proteins and their biotechnological production could open new avenues for natural sweetener development. As this study highlights, the convergence of sensory science and biotechnology will be essential for identifying and commercializing next-generation sweeteners that align with public health goals.

While developing this project, we also recognized that the use of recombinant DNA technology, fusion proteins, and subsequent cleavage steps in the production of brazzein may raise concerns regarding the perception of genetically modified organisms (GMOs), particularly in EU market where regulatory frameworks and public sentiment towards GMOs are more restrictive. However, it is important to note that the primary focus of our work was to develop a scalable and efficient biotechnological process for brazzein production, leveraging well-established techniques in protein engineering. While this approach involves the use of GMOs, the final product brazzein is a purified protein, identical in structure and function to its natural counterpart found in nature. Furthermore, the technology developed here has the potential to be used for the production of other pharmaceutical peptides or small proteins.

We also acknowledge the importance of public perception and the need for transparent communication about the use of biotechnological methods in food production. To address potential market limitations, future research could explore alternative production methods, such as cell-free protein synthesis or plant-based expression systems, which may be more acceptable to consumers wary of GMOs.

In conclusion, this study advances our understanding of brazzein production and demonstrates the power of biotechnological innovation in addressing public health challenges associated with sugar consumption. The methodologies developed here have the potential to catalyze the production of a wide range of functional proteins, contributing to the fields of food science, nutrition, and beyond.

## Supplemental Materials

Supplementary data for this paper are available on-line only at http://jmb.or.kr.
