# Supplementary material for: Novel gnd_v2 Fusion Tag and Engineered TEV Protease Enable Efficient Production of Brazzein
Source: J Microbiol Biotechnol. 2024 Sep 9;34(11):2310–20. doi: 10.4014/jmb.2407.07047 (PMC11637865; doi:10.4014/jmb.2407.07047)
Supplement: Supplementary file 1 [file jmb-34-11-2310-supple.pdf]

J. Microbiol. Biotechnol. 2024. 34(11): 2310–2320
https://doi.org/10.4014/jmb.2407.07047

Supplementary Figures and Table

Optimization of a 6-Phosphogluconate Dehydrogenase (gnd) Fusion Tag for Soluble Expression and Streamlined Purification of the Sweet Protein Brazzein

Yu Wang[1], Jiayao Zheng[1], Fan Wen[1], Bowen Tu[2], and Lun Cui[1,*]

[1] CCZU-JITRI joint Bio-X Lab, School of Pharmacy & School of Biological and Food Engineering, Changzhou University, 213164, Changzhou, Jiangsu Province, China
[2] Pathogenic Biological Laboratory, Changzhou Disease Control and Prevention Centre, Changzhou Medical Centre, Nanjing Medical University, 213000, Changzhou, Jiangsu Province, China

*Corresponding Author(s), E-mail(s): luncui@cczu.edu.cn
Contributing authors, E-mail: Yu Wang: WY17802585208@outlook.com
Jiayao Zheng: s22090860003@smail.cczu.edu.cn
Fan Wen: wenfan25@outlook.com
Bowen Tu: tbwchangzhou@163.com

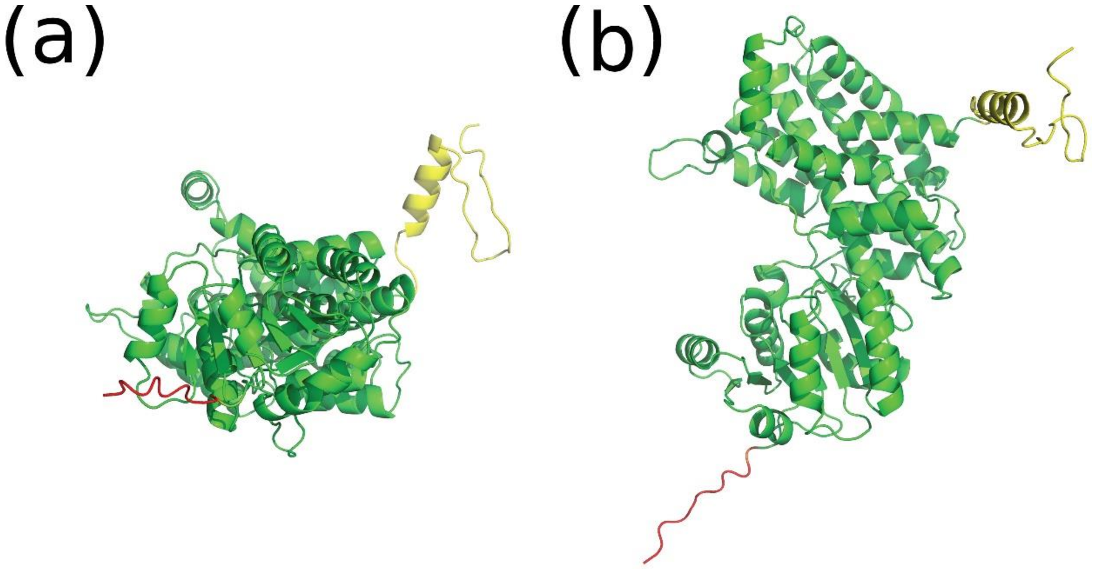

Fig. 1. Predicted Structural of his-gnd. (a) Side view of the his-gnd. The middle core part of his-gnd tag is depicted in green, highlighting its globular form. The his tag was shown in red, the c-terminal part was shown in yellow. **(b)** Top view (90-degree rotation from panel a) of the his-gnd structure, offering a different perspective on the position of his tag and c-terminal part relative to the gnd core part.

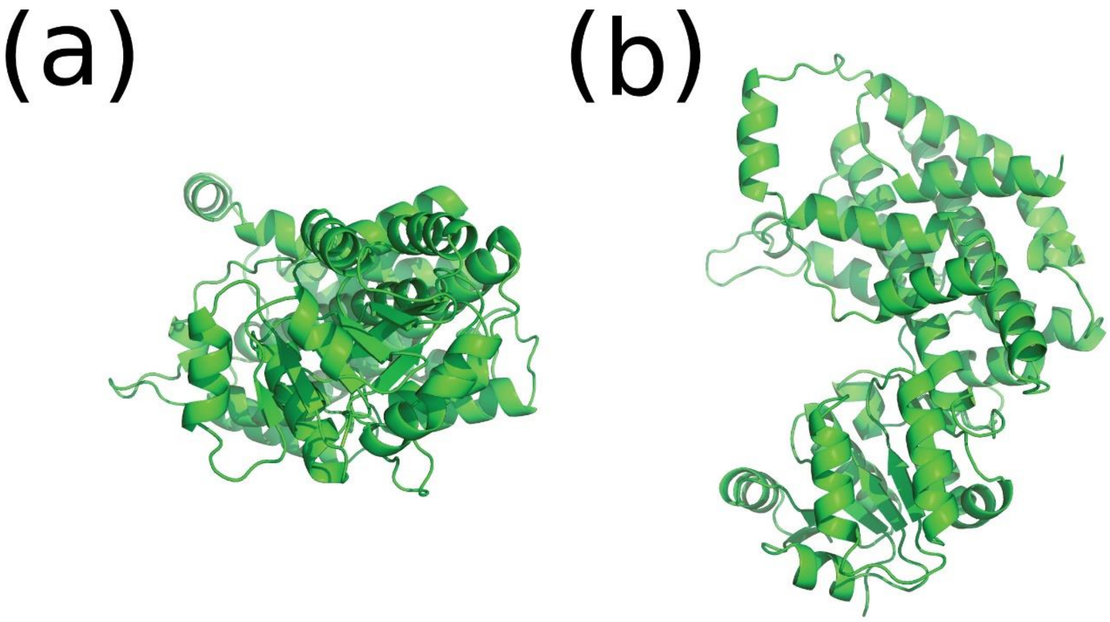

Fig. 2. Predicted 3D Structure of the gnd_v2 Tag. (a) The three-dimensional structure of the gnd_v2 tag as observed from the side. This view illustrates the folding patterns and spatial configuration of the tag, with alpha-helices and beta-sheets prominently displayed in globular form. **(b)** The same gnd_v2 tag structure as in panel **(a)**, rotated 90 degrees.

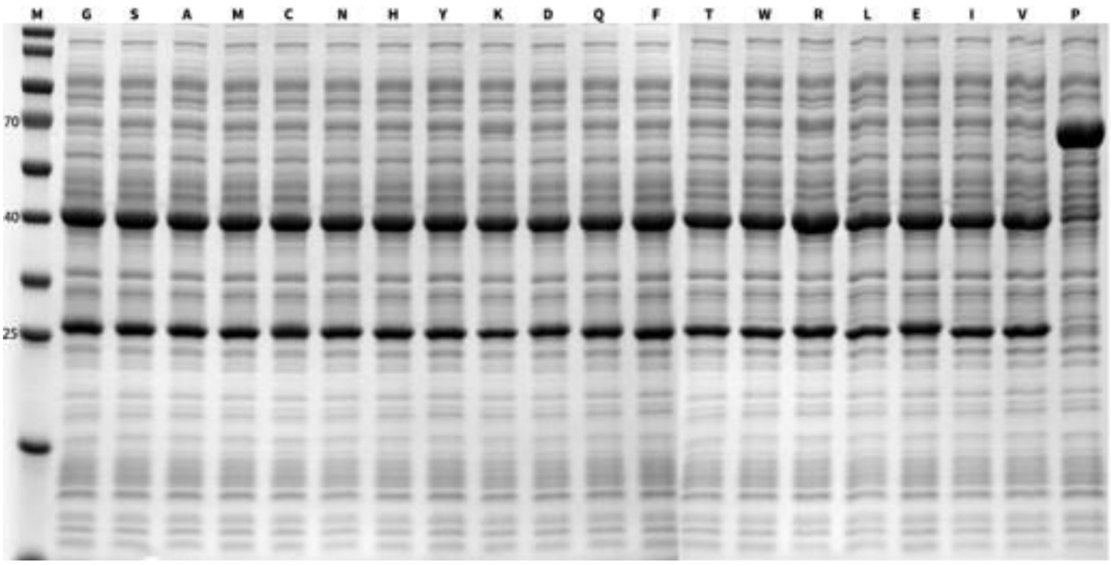

Fig. 3. Cleavage Spectrum Analysis of TEV Max Protease. This gel electrophoresis image presents the cleavage efficiency of TEV Max protease across a spectrum of 19 amino acids at the P1' site. Each lane corresponds to a single amino acid residue, represented by their one-letter codes (G for Glycine, S for Serine, etc.), showing protease activity results. The consistent pattern of bands across the lanes demonstrates TEV Max's broad substrate specificity and ability to efficiently cleave at all tested amino acids, except for Proline (P, in the last lane), as indicated by the absence of a cleaved corresponding band.

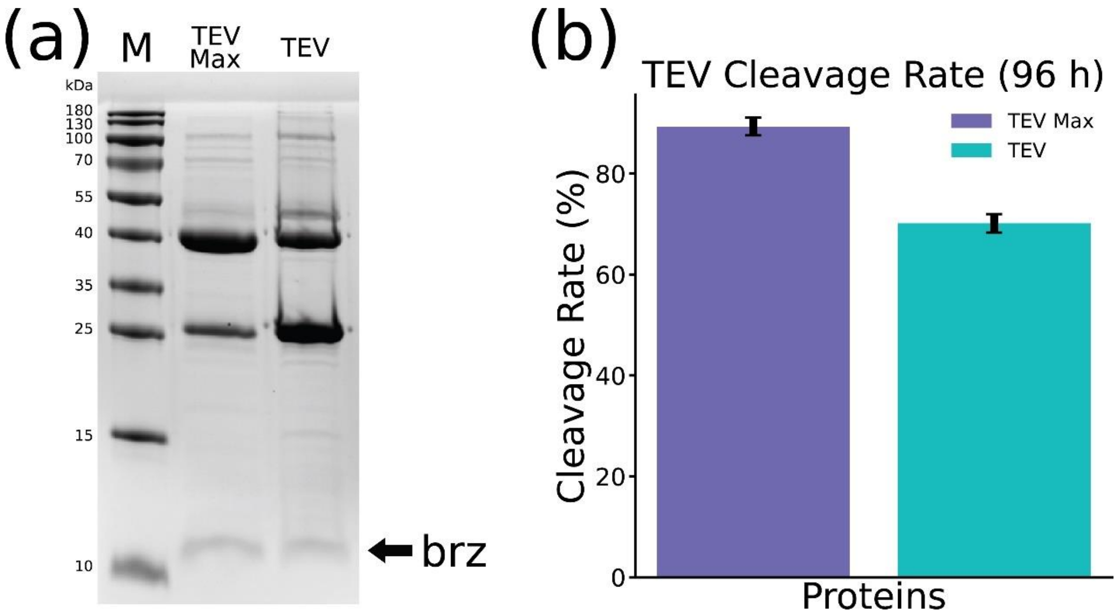

Fig. 4. Cleavage Efficiency Comparison Between TEV Max and Wild-Type TEV (96 h)

(a) SDS-PAGE analysis of brazzein (brz) cleavage by TEV Max and wild-type TEV proteases after a 96-hour incubation period. Lane M displays the molecular weight markers. The 'TEV Max' lane shows the cleavage products following treatment with TEV Max protease, while the 'TEV' lane shows those following treatment with wild-type TEV protease. The arrow indicates the band corresponding to the cleaved brazzein protein. **(b)** Bar graph depicting the cleavage rate (%) of TEV Max (purple) versus wild-type TEV (cyan) proteases. Error bars represent the standard deviation from replicate assays (n=3).

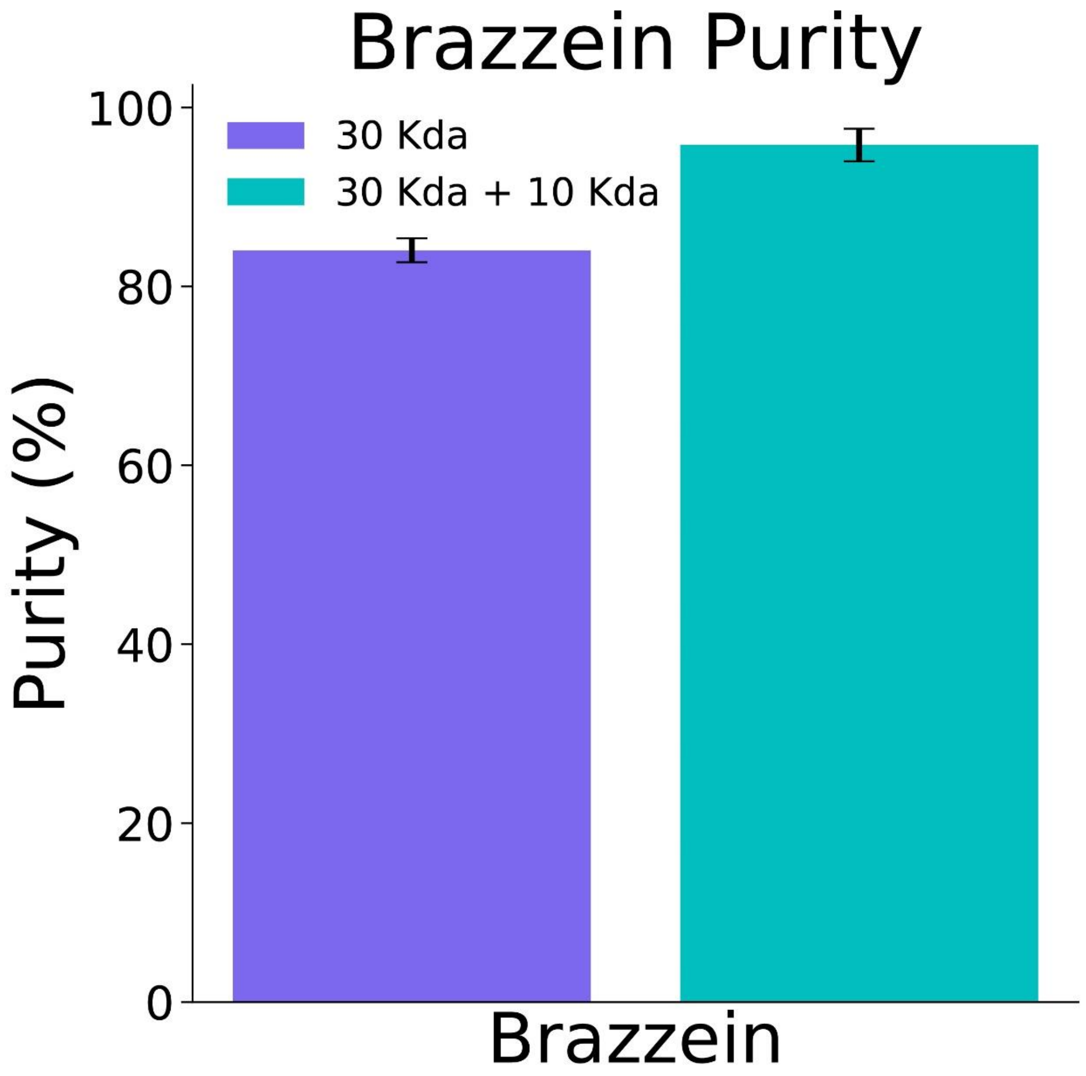

Fig. 5. Brazzein Purity Post-Ultrafiltration Steps. This bar graph illustrates the purity levels of brazzein after sequential ultrafiltration steps based on SDS-PAGE gel analysis. The purple bar represents the purity percentage after the initial ultrafiltration with a 30 kDa molecular weight cut-off (MWCO) filter. The cyan bar indicates the enhanced purity achieved after a subsequent ultrafiltration step using a 10 kDa MWCO filter. Error bars denote the standard deviation from multiple analyses (n=3).

Table 1: Primer Information.

Primer Name	Sequences (5'→3')
WY67	GAAAATCTTTATTTTCAACAAGATAAATGTAAAAAGGTATATG
WY68	TTGAAAATAAAGATTTTCGTCCAGCCATTCGGTATGAA
WY88	ATGGGCAGCAGCCATCACCATCATCACCACACAGATGTAACGATTAAAACGCTG
WY93	ACATTTATCTTGTTGAAAATAAAGATTTTCGCTCACTTTGTCTTTTTCCGC
WY97	ACATTTATCTTGTTGAAAATAAAGATTTTCCGTGGAATAGGTCGCGCC
WY116	TAATAAGGAGATATACCATGTCAAAACAGCAAATAGGGGTAG
WY131	TTGAAAATAAAGATTTTCTGCTGCGCGGTAGCTATCA
CYJ150	GACCTGCAGGCGCGCCGTTAATATTCGCAGTAATCGCAGATG
CYJ151	TAACGGCGCGCCTGCAGGTC
CYJ158	GCCATCACCATCATCACCACTCAGATAGCGAAGTG
CYJ170	GCCATCACCATCATCACCACTCAAAACAGCAAATAGG
LC368	CATGGTATATCTCCTTATTAAAGTTAAAC
LC908	GTGGTGATGATGGTGATGGC

Supplementary sequence 1 (Synthesized brz fragment):

CAAGATAAATGTAAAAAGGTATATGAAAACTACCCGGTTTCCAAGTGCCAGTTGGCGAACC
AGTGTAATTACGACTGTAAACTGGATAAACACGCTCGTAGCGGTGAGTGCTTCTATGACGA
GAAGCGCAACCTGCAATGCATCTGCGATTACTGCGAATATTAA

Supplementary sequence 2 (Synthesized TEV Max fragment):

GGAGAATCACTATTTAAAGGGCCGAGGGATTACAACCCGATCAGCAGCAGTATTTGCCATT
TGACCAACGAGAGCGACGGCCACACCACCTCACTGTACGGCATCGGCTTTGGTCCGTTTAT
CATCACCAACAAACACTTGTTTCGCCGTAATAACGGTACGCTGCTGGTTCAGTCCCTTCACG
GCGTTTTTAAAGTTAAGGACACTACGACTTTACAGCAACATTTGGTTGATGGGAGAGACAT
GATTATCATCCGCATGCCGAAGGACTTTCCACCGTTCCCGCAAAAACTGAAATTCCGCGAG
CCGCAACGTGAGGAACGTATTTGCCTGGTCACCACGAACTTCCAAACCAAATCCATGTCTT
CTATGGTGTCCGATACCAGCTGCACCTTCCCAAGCGGTGATGGTACATTTTGGAAACATTG
GATTCAGACCAAGGACGGCCAGTGTGGTAATCCGCTGGTGTCTACCCGTGATGGCTTCATC
GTGGGTATTCACAGCGCGAGCAATTTCACCAACACCAACAACTATTTCGCGAGCGTTCCGA
AGAACTTTATGGAACTGCTGACGAACCAAGAAGCTCAGCAGTGGGTTTCCGGCTGGCGTCT
CAATGCAGATAGCGTGCTGTGGGGTGGTCACAAAGTGTTCATGAACAAGCCGGAAGAACC
ATTCCAGCCGGTTAAGGAGGCTACCCAGCTGATGAAT
